# Interactions of Archaeal Chromatin Proteins Alba1 and Alba2 with Nucleic Acids

**DOI:** 10.1371/journal.pone.0058237

**Published:** 2013-02-28

**Authors:** Miha Črnigoj, Zdravko Podlesek, Mateja Zorko, Roman Jerala, Gregor Anderluh, Nataša Poklar Ulrih

**Affiliations:** 1 Biotechnical Faculty, University of Ljubljana, Ljubljana, Slovenia; 2 National Chemical Institute of Slovenia, Ljubljana, Slovenia; 3 Faculty of Chemistry and Chemical Technology, University of Ljubljana, Ljubljana, Slovenia; 4 Centre of Excellence EN-FIST, Ljubljana, Slovenia; 5 Centre of Excellence for Integrated Approaches in Chemistry and Biology of Proteins (CipKeBiP), Ljubljana, Slovenia; The Scripps Research Institute, United States of America

## Abstract

**Background:**

Architectural proteins have important roles in compacting and organising chromosomal DNA. There are two potential histone counterpart peptide sequences (Alba1 and Alba2) in the *Aeropyrum pernix* genome (APE1832.1 and APE1823).

**Methodology/Principal Findings:**

These two peptides were expressed and their interactions with various DNAs were studied using a combination of various experimental techniques: surface plasmon resonance, UV spectrophotometry, circular dichroism–spectropolarimetry, gel-shift assays, and isothermal titration calorimetry.

**Conclusions/Significance:**

Our data indicate that there are significant differences in the properties of the Alba1 and Alba2 proteins. Both of these Alba proteins can thermally stabilise DNA polynucleotides, as seen from UV melting curves. Alba2 and equimolar mixtures of Alba1/Alba2 have greater effects on the thermal stability of poly(dA-dT).poly(dA-dT). Surface plasmon resonance sensorgrams for binding of Alba1, Alba2, and equimolar mixtures of Alba1/Alba2 to DNA oligonucleotides show different binding patterns. Circular dichroism indicates that Alba2 has a less-ordered secondary structure than Alba1. The secondary structures of the Alba proteins are not significantly influenced by DNA binding, even at high temperatures. Based on these data, we conclude that Alba1, Alba2, and equimolar mixtures of Alba1/Alba2 show different properties in their binding to various DNAs.

## Introduction

The Alba (acetylation lowers binding affinity) proteins are part of a protein superfamily that spans all three domains of life. For the most part, however, their functions remain obscure. The Alba proteins were first identified as components of chromatin in the thermoacidophilic archaeon *Sulfulobus*, although they were postulated to have additional functions due to their structure and their binding to RNA [1]. The Alba superfamily of proteins has been split into three major branches. One is the archaeal branch, which is typified by proteins such as *Sulfulobus shibatae* SsH10b. The other two branches are eukaryote specific, and these are exemplified by the human and yeast RNase/MRP subunits Rpp20/Pop7 (Alba1 and Alba2) and Rpp25/Pop6 (Alba3 and Alba4), with the latter including the ciliate protein Mdp2 [2]. Alba domain proteins appear to have had great functional plasticity through the course of evolution. Following their first identification as DNA-binding proteins in Archaea, they were found in association with the nuclear RNase MRP/P in yeast and mammalian cells [2].

All known hyperthermophilic Archaea contain at least one type of Alba protein. They are the second most abundant chromatin proteins in Archaea, and are probably at least partially responsible for DNA stabilisation in hyperthermophiles [3]. The Alba proteins are basic and they bind to DNA in a cooperative manner, although with no apparent sequence specificity. There are 37 Alba proteins known in Archaea [4]. *Pyrobaculum aerophylum* and *Aeropyrum pernix* are hyperthermophiles where the Alba proteins appear to be their main DNA-binding proteins [5], [6]. In these two hyperthermophiles, the Alba proteins account for a considerable portion of cell-protein synthesis (up to 5%), and it is believed that the synthesis of the Alba1 protein is about 20-fold greater than that of Alba2 [7].

In solution, the Alba proteins are usually dimers that are formed from subunits that are approximately 10 kDa in size, which are primarily stabilised by hydrophobic interactions. Each dimer has two antiparallel β-hairpins that protrude out of the main globular structure, which have been proposed to be responsible for binding to the minor DNA groove [4]. It has been shown by isothermal titration calorimetry (ITC) and nuclear magnetic resonance that the Alba proteins form homodimers and heterodimers [8]. Electron microscopy of DNA-bound Alba proteins has revealed that lower Alba concentrations allow Alba to connect across two DNA molecules; however, with higher concentrations of Alba, a highly condensed Alba/DNA structure is formed [8], [9]. Additionally, at lower concentrations, the Alba monomers cover 12 bp dsDNA, as opposed to 6 bp when higher concentrations of Alba are used. It has been demonstrated that the Alba1/Alba2 heterodimer promotes the organisation of chromatin at higher levels [4], [7], [8]. The Alba proteins bind with the same affinity to dsDNA fragments of different lengths, which indicates that acetylation of Alba proteins does not directly influence their affinity for DNA, although this probably represents a molecular signal for other proteins [8]. Recently, the high resolution structure of the Alba2-dsDNA (16 bp) complex from *A. pernix* was reported [10]. The overall structure of this complex reveals a discrete mode of DNA binding, with the positively charged residues on the monomer-monomer interface of each dimer packing into the minor groove of the bound dsDNA. However, only 4 bp of the dsDNA was ordered in the structure with the Alba2 dimer for this particular complex, which prevented potential interactions of the β3–β4 turn of Alba2 with the DNA. Nevertheless, based on the interactions between the proteins in adjacent asymmetric units, the authors proposed that the array of proteins packs above and below the dsDNA.

The aim of the present study was to investigate the binding of the Alba1 and Alba2 proteins from *A. pernix* to various dsDNAs at 25°C, through the use of surface plasmon resonance (SPR) and ITC, and to study the effects of the Alba proteins on the thermal stability of the DNA by following the helix-coil transition by UV spectrophotometry. Furthermore, we investigated the conformational changes of the Alba proteins after their binding to the DNA by circular dichroism (CD) spectropolarimetry.

## Experimental Procedures

### Purification of the Alba Proteins

The Alba proteins were cloned into either pQE-30UA *Escherichia coli* M15 (Alba1), or pETDuet-1 *E. coli* BL21 (DE3) pLysS (Alba2), both with a His-tag (removable with thrombin cleavage). Additionally, Alba2 was co-expressed with non-His-tagged Alba1 to achieve better solubility of the protein. *E. coli* was cultivated at 46°C after activation of synthesis with 1 mM isopropyl-beta-D-thiogalactopyranoside. The proteins were purified as described previously [11], by combination of (NH_4_)_2_SO_4_ precipitation, NiNTA, ultrafiltration, and temperature-induced precipitation of impurities. Protein concentrations were determined with the Bio-Rad (Biorad, USA) and bicinchoninic acid (BCA; Pierce, USA) protein assays, with the BCA protein assay showing more accurate protein concentrations.

### Agarose Gel Mobility Shift Assay

Agarose gel mobility shift assays (EMSAs) were used to examine the effects of binding of Alba1 and Alba2 with or without the His-tag to linear DNA molecules [11], [12]. A 2686 bp linearised DNA (pUC19/*Eco*RI) was used. All DNA/Alba protein mixtures were incubated for 1 h at room temperature (or at 50°C) prior to the EMSAs, which were carried out with 0.8% agarose gels.

### Polyacrylamide Gel Electrophoresis

Electrophoresis was performed on the native proteins with 20% acrylamide gels on a PhastSystem (Sweden). Acidic buffer agarose strips were used, with the proteins migrating towards the negative electrode (reverse electrode). We used the well-characterised equinatoxin II for size comparison (19.82 kDa, pI 10.5), which has similar properties to the Alba proteins [13]. SDS-electrophoresis was performed without and with the reducing agent (83 mM dithiothreitol) in 22% SDS acrylamide gels, to establish the oxidative/reductive states of the –SH groups in the Alba proteins.

### Surface Plasmon Resonance

Biotinylated DNA oligonucleotides (35 nucleotides in length) were immobilised to the SPR sensor chips (SA; Biacore; GE Healthcare). All of the measurements were carried out on a Biacore T100 instrument. The dsDNA oligonucleotides had seven nucleotide GC clamps at both ends of the sequences, to ensure the double stranded conformation of the molecule (TIB Molbiol, Germany). For each oligonucleotide, only one strand was biotinylated at the 5′-end, to avoid multiple binding of a single oligonucleotide to the SPR sensor chip. Central sequences (21 nucleotides) were designed with the aim of random sequences based on the *A. pernix* genome (SPRspecAP; 5′-Biot-CCCCCCCGTGAAAGCCTAGACAGCGAGGCCCCCCC-3′). The DNA oligonucleotides were added to the sensor chip until the immobilisation level of 1000 resonance units (RU) was reached. The analyte was injected over the ligand immobilised on the sensor chip, in 50 mM NaH_2_PO_4_, 150 mM NaCl, 0.005% P20, pH 7.0 and at 25°C. The flow rate was 5 µL min^−1^, with association for 7 min and dissociation for 6 min. The analytes used were Alba1, Alba2, Alba1/Alba2, and bovine serum albumin (as the negative control). The sensor chip was regenerated after the analyte binding using three 60-s injections of 2 M NaCl at a flow rate of 5 µL min^−1^.

### UV Spectrophotometry

The UV light absorbance *versus* temperature was measured using a UV-vis spectrophotometer (Cary Varian Cary 100 Bio, Australia) equipped with a thermoelectrically controlled cell holder. The UV melting curves were obtained by UV absorption measured as a function of temperature, for the various DNA polynucleotides: calf thymus DNA (CT-DNA); poly(dA-dT).poly(dA-dT) (AT-DNA); and poly(dG-dC).poly(dG-dC) (GC-DNA). This was carried out in the presence of different molar ratios of Alba1 and Alba2, and an equimolar mixture of Alba1/Alba2. The measurements were at 260 nm in the temperature range from 20°C to 99°C, with a heating rate of 1°C min^−1^ and an equilibration time of 1 min. The concentration of double-stranded polynucleotides was determined spectrophotometrically using the following molar extinction coefficients expressed as molar concentrations of base pairs: calf thymus DNA, ε_259_ = 12,800 M^−1^ cm^−1^; poly(dA-dT).poly(dA-dT), ε_260_ = 13,300 M^−1^ cm^−1^; poly(dG-dC).poly(dG-dC), ε_254_ = 16,800 M^−1^ cm^−1^. These values were either provided by the manufacturer or taken from the literature [14]. The concentration of the DNA per base pairs in the experimental cuvettes was always 10 µM. The thermal denaturation of Alba1 and Alba2 was measured in the temperature range from 20°C to 99°C at 280 nm, at molar ratios of protein/DNA base pair from 1∶30 to 1∶2. The final Alba (monomer) concentrations in the experimental cuvettes were between 0.333 µM and 5.0 µM.

### Circular Dichroism

The CD spectra of Alba1 and Alba2 and the various DNAs were measured using an AVIV Model 62A DS spectropolarimeter (AVIV Associates, Lakewood, NJ), equipped with a thermoelectrically controlled cell holder. Cuvettes with path lengths of 1 mm were used for far-UV (200–260 nm). The Alba1, Alba2 and Alba1/Alba2 protein concentrations were 0.2 mg·mL^−1^ in 50 mM NaH_2_PO_4_, pH 7.0. The various DNAs in the mixture (CT-DNA, AT-DNA, GC-DNA) were added to a final Alba:DNA base pair molar ratio of 1∶5. The CD spectra were recorded as a function of temperature (at 25, 50, 70 and 90°C), and all of the reactions were incubated for 20 min at room temperature prior to the measurements. The mean residue ellipticity, [Θ]_λ_, was calculated using the relationship:
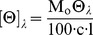
(1)where M_o_ is the mean residue molar mass (109.96 g mol^−1^ for Alba1, and 111.57 g mol^−1^ for Alba2), Θ_λ_ is the measured ellipticity in degrees, c is the concentration in g mL^−1^, and l is the path length in decimetres. [Θ]_λ_ was expressed in deg cm^2^ dmol^−1^. The secondary structure content was calculated from the far-UV CD spectra using the CONTIN software package [15]. The degree of reversibility in the temperature-induced unfolding of Alba1 and Alba2 was determined by measuring the CD spectrum at 25°C after heating the proteins to 90°C. The CD spectra of DNA at the corresponding temperatures were subtracted from all of the CD spectra of the Alba proteins incubated with the DNA.

### Isothermal Titration Calorimetry

The heat flow that resulted from the binding between Alba1 and Alba2 and the DNA oligonucleotides was measured using a high-sensitivity MicroCal isothermal titration calorimeter (VT-ITC MicroCalorimeter) in a reaction cell (1.4 mL) at a stirring speed of 350 rpm. In each titration, the reaction cell was loaded with the various DNAs (30 µM [bp] for CT-DNA, 56 µM [bp] for the A-DNA (CCCGGGCCCGGGCCCGGGCCCGGGCCCGGGCCCGGGCCCGGGCCCGGG) and the B-DNA (ACGTACGTACGTACGTACGTACGTACGTACGTACGTACGTACGTACGT) and for the sequences of 20–30 successive 2 µl to 10 µl aliquots, the injections were performed using a 250 µl auto-syringe filled with Alba1 solution (300 µM or 150 µM), with 2 min between each injection. To correct for the heat effects of dilution and mixing, control experiments were performed at the some concentration of protein. The calorimetric data were analysed and converted into enthalpy changes using the MicroCal Origin 7.0 software provided with the instrument. The enthalpy change for each injection was calculated by integrating the area under the peaks of the recorded time, and then correcting with the control titration. The experimental data were fitted to a binding model using a non-linear least squares method, with n, ΔH_bin_ and K_bin_ as adjusted parameters. The experiments were performed at 25°C and pH 7.0 (50 mM NaH_2_PO_4_).

### Molecular Modelling

The Alba proteins were docked onto the DNA using PatchDock [16]. Binding of Alba to the DNA at high density was performed by subsequent docking of additional Alba proteins into the nearest chemically identical environment, which prevented steric clashes with the bound Alba protein. The conformations of Alba2 and Alba1 were taken from the PDB entries 3U6Y, 2H9U and 2BKY.

## Results

### Characterisation of the Alba Proteins

**Figure 1:**
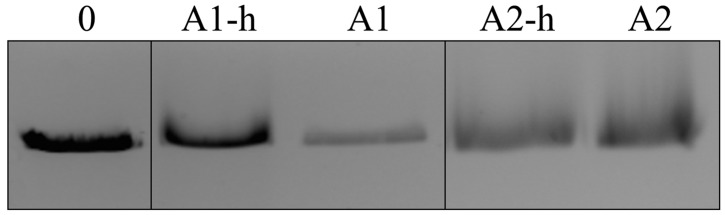
Electrophoretic mobility shift assay of Alba1 and Alba2 proteins binding to linear DNA (pUC19/*Eco*RI). The Alba proteins with the His-tag are marked as A1-h and A2-h and without the His-tag as A1 and A2. Lane 1: linear DNA (pUC19/*Eco*RI) in the absence of the Alba proteins. Lanes 2–5: DNA with and without the His-tagged Alba proteins, as indicated.

**Figure 2:**
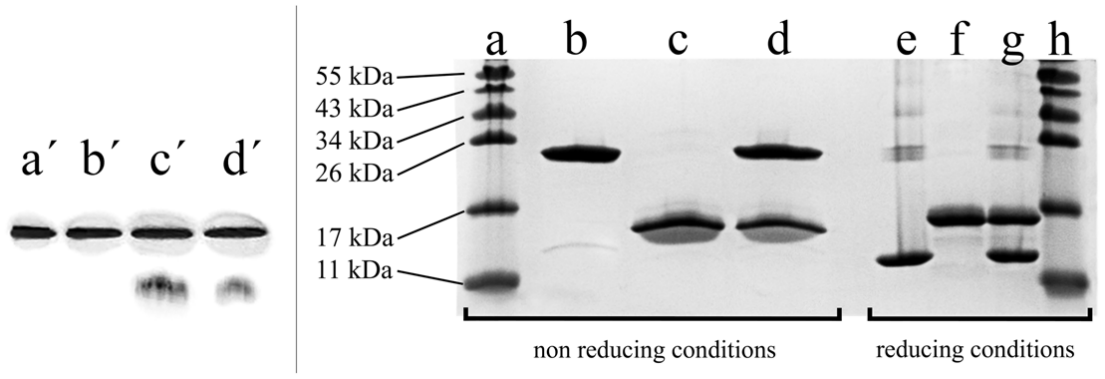
Native and SDS PAGE electrophoresis. Left: Native protein PAGE electrophoresis with 20% homogeneous gel for the Phast system. á, equinatoxin II; b´, Alba1; c´, Alba2; and d´, equimolar ratio of Alba1/Alba2. Right: SDS PAGE electrophoresis shows influence of disulphide bridges (±DTT) on dimerisation of Alba proteins. a, h, PageRuler protein ladder; b, e, Alba1; c, f, Alba2; d, g, equimolar ratio of Alba1/Alba2.

There are two known DNA sequences for the Alba proteins in the Archaea *A. pernix*: APE1832.1 (Alba1; Ape10b1) and APE1823 (Alba2; Ape10b2). Alba1 is composed of 94 amino-acid residues (10,336 Da) and has a theoretical isoelectric point (pI) of 9.5. Alba2 has 102 amino-acid residues (11,380 Da) and a theoretical pI of 9.1 [4], [8]. Both of the Alba proteins bound to the DNA in EMSAs regardless to the presence of a His-tag (removed via a thrombin cleavage site) (Figure 1). Similarly, no differences in DNA shift were observed if the Alba protein/DNA mixtures were incubated at higher temperatures (50°C) prior to EMSA. In the native protein acrylamide gel electrophoresis, the Alba proteins co-migrated with equinatoxin II (19.82 kDa, pI 10.2), which was used as a reference. Under the applied conditions, Alba1 appeared as a single band in the gel, while Alba2 and the equimolar mixture of both of these Alba proteins showed two bands, where the weaker band is likely to correspond to a monomer, and the stronger band to a dimer (Figure 2, left side).

In the non-reducing SDS PAGE (20%), the Alba1 protein migrated as a 24 kDa protein (Alba1 dimer); a very weak band was observed at approximately 13 kDa (Figure 2, right side, lanes b-d). Under the reducing conditions (dithiothreitol added), Alba1 migrated as a protein of 13 kDa (Figure 2, right side, lanes e-g), which corresponded to the migration of the weak 13 kDa band in the non-reducing SDS PAGE. Under reducing conditions, weak multiple bands of higher molecular masses were observed (24 kDa, 34 kDa and 43 kDa) (Figure 2, lane e), which indicated strong interactions between the Alba1 monomers or the rapid reformation of the dimers after the removal of DTT. Alba2 migrated in the reducing and non-reducing SDS PAGE as a protein of 15 kDa (Figure 1, lanes c, f). When an equimolar mixture of Alba1/Alba2 (1∶1) was used (under reducing and non-reducing conditions), two strong bands were observed (13 kDa and 15 kDa), which corresponded to Alba1 and Alba2 (Figure 1, right side, lanes d, g). The weaker bands seen in Figure 2 lanes e and g correspond to Alba1 oligomers, which were probably formed because of hydrophobic interactions and due to the lack of a stabilising effect of intermolecular disulphide bonds in the reduced state of the protein.

Native protein electrophoresis indicated that Alba1, Alba2 and the equimolar mixture of Alba1/Alba2 are found as dimers in solution (Figure 2, left). The net charge of the proteins at pH 7.0 was 3+ for Alba1 and 1+ for Alba2, which are therefore comparable with the equinatoxin II protein, which was used as the standard, and which had a net charge of 2+ at pH 7.0. The different electrophoretic mobilities can be ascribed mainly to the sizes of the molecules. The molecular weights of the Alba proteins are approximately half that of our reference protein equinatoxin II. The data from the gel electrophoresis indicate that the Alba proteins probably appear as dimers in solution. Our findings coincide with data of Alba homologues from other hyperthermophilic Archaea [4], [7], [8], [17], [18]. Previously, the formation of the Alba1/Alba2 heterodimer was shown by NiNTA agarose chromatography in which only one of two *Aeropyrum pernix* Alba proteins was His-tagged, but both proteins co-eluted [11], as was reported for the Alba proteins from *Sulfolobus solfataricus* [8].

### Binding Studies of DNA/Alba Interactions Detected by Surface Plasmon Resonance

**Figure 3:**
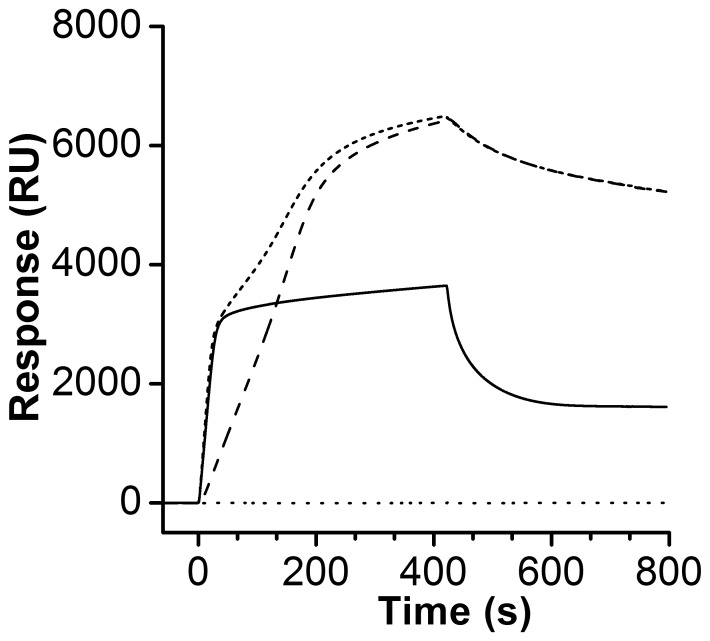
Surface plasmon resonance sensorgram of the binding of the Alba proteins to the SPRspecAP oligonucleotide. The Alba protein concentration was 6.0 µM. Alba1 (–), Alba2 (·□···□·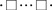), the Alba1/Alba2 complex (­­­), and bovine serum albumin (···) at 25°C.

Surface plasmon resonance revealed that both of the Alba proteins bind the DNA oligonucleotides in a complex manner (Figure 3). Bovine serum albumin was used as a negative control, and it showed no DNA binding in all cases. Alba1 showed saturable binding under the conditions used, with fast association. Alba2 showed slower association, and also dissociation, while the sensogram of the Alba1/Alba2 equimolar complex with the SPRspecAP oligonucleotide showed a biphasic pattern of binding to DNA (Figure 3).

### The Effects of the Alba Proteins on the Thermal Stability of DNA

The effects of the Alba1 and Alba2 proteins on the thermal stability of the different DNAs (CT-DNA, AT-DNA, GC-DNA) was evaluated spectrophotometrically.

**Figure 4:**
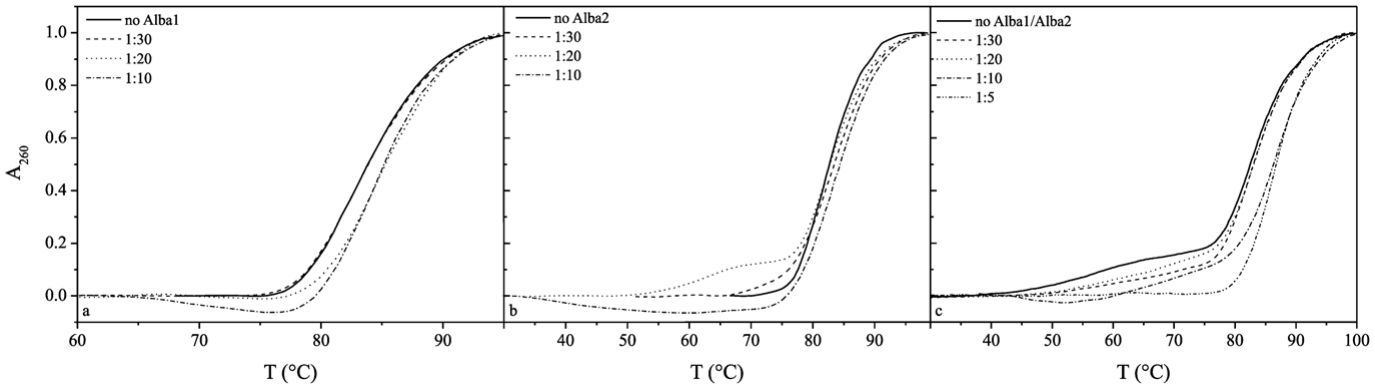
UV melting curves of CT-DNA. The molar ratios of protein:DNA base pairs were, at 1∶30 to 1∶10 for Alba1 (a) and Alba2 (b), and at 1∶30 to 1∶5 for the Alba1/Alba2 complex (c).

**Figure 5:**
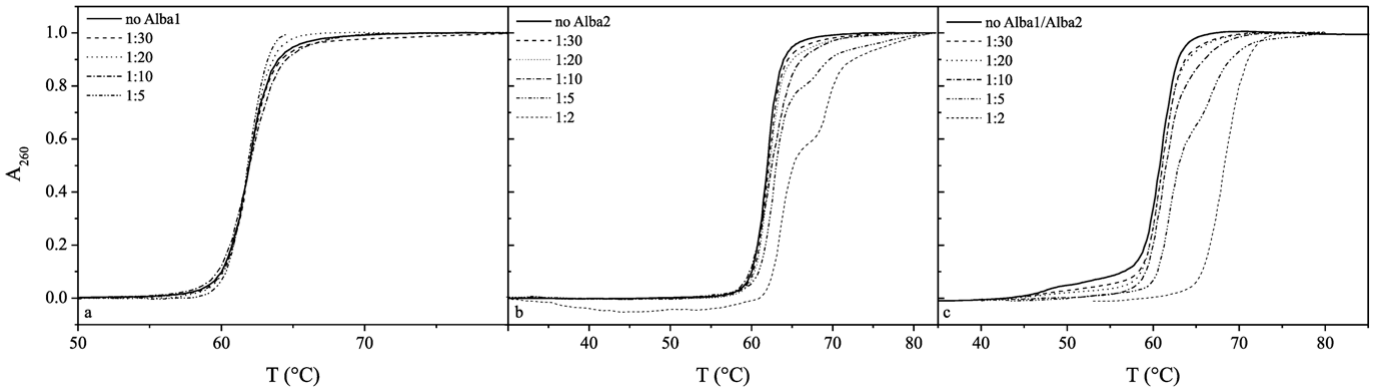
UV melting curves of AT-DNA. The molar ratios of protein:DNA base pairs were at 1∶30 to 1∶5 for Alba1 (a) and at 1∶30 to 1∶2 for Alba2 (b) and the Alba1/Alba2 complex (c).

**Figure 6:**
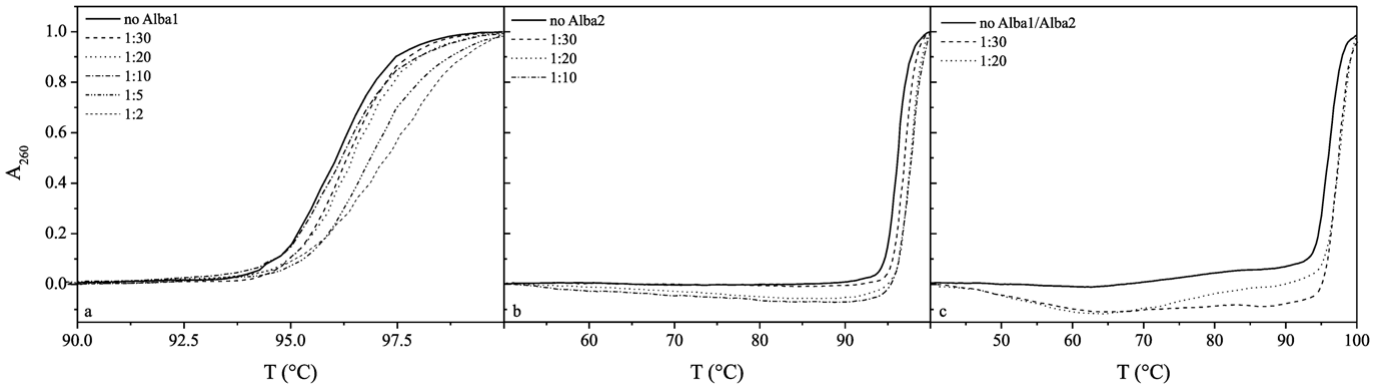
UV melting curves of GC-DNA. The molar ratios of protein:DNA base pairs were at 1∶30 to 1∶2 for Alba1 (a), at 1∶30 to 1∶10 for Alba2 (b), and at 1∶30 to 1∶20 for the Alba1/Alba2 complex (c).

The effects of Alba1 and Alba2 and the equimolar mixture of Alba1/Alba2 on the thermal profiles of these DNAs are shown in Figures 4, 5 and 6. The melting temperature (T_m_) of CT-DNA at pH 7.0 (50 mM NaH_2_PO_4_) in the absence of the proteins was 83°C, and this increased to 86°C at the molar ratio of 1∶5 of Alba1:CT-DNA base pairs (Table 1). Although only a small thermal stabilisation effect was seen here (ΔT_m_ *ca*. 3°C), a significant condensation effect of Alba2 on CT-DNA was seen at molar ratios of 1∶10 and higher (Figure 4b). The addition of the Alba1 protein to AT-DNA had no significant effects on its thermal stability, although condensation of AT-DNA was seen at molar ratios higher than 1∶5 (Figure 5a). Similarly, the addition of the Alba1 protein to GC-DNA had no significant thermal stabilisation effects on the DNA, as the difference in the T_m_ at Alba1:GC-DNA molar ratios up to 1∶20 was only 1.5°C (Figure 6a).

**Table 1:** Apparent binding constants (K_bin_) at 1∶5 molar ratios of the Alba proteins and CT-DNA, AT-DNA and GC-DNA, determined at the T_m_.

	T_m_	ΔT	K_bin_
	(°C)	(°C)	(M^–1^)
**Alba1**			
CT-DNA	85.5±0.5	2.5±1.0	3.7 ×10^5^
AT-DNA	62.0±0.5	0.3±1.0	2.6 ×10^5^
GC-DNA	96.8±0.5	0.8±1.0	5.5 ×10^5^
**Alba2**			
CT-DNA	85.0±0.5	2.0±1.0	3.7 ×10^5^
AT-DNA*	68.0±0.5	6.3±1.0	6.9 ×10^5^
GC-DNA	100.0±0.5	4.0±1.0	1.2 ×10^6^
**Alba1/Alba2**			
CT-DNA	86.5±0.5	3.5±1.0	5.9 ×10^5^
AT-DNA	67.0±0.5	5.3±1.0	1.2 ×10^6^
GC-DNA	99.3±0.5	3.3±1.0	2.6 ×10^6^

* The transition is not a two-state one.

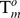
 is the melting temperature of the nucleic acid duplex in the absence of Alba proteins, and for CT-DNA, AT-DNA and GC-DNA it is 83.0°C, 61.7°C and 96.0°C, respectively. 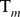 is the melting temperature of the Alba-DNA complex at the R value of 0.2 determined from the ITC (Table 2). ΔT ( = 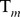
–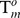). Kbin values were calculated using Eq. 2, as described in the text. For the application of Eq. 2, the following calorimetrically determined ΔHWC values for the melting of a base pair were used: ΔHWC = 8.5 kcal/mol base pair for calf thymus DNA; ΔHWC = 7.6 kcal/mol base pair for poly(dA-dT).poly(dA-dT), and ΔHWC = 14.7 kcal/mol base pair for poly(dG-dC).poly(dG-dC) [20]. Buffer conditions: pH 7.0 (50 mM NaH2PO4). Standard errors in Kbin calculation were lower than 5%.

Addition of Alba2 to CT-DNA (T°_m_, 83°C) slightly destabilised the CT-DNA at the lower molar ratios (1∶30 and 1∶20) and stabilised it at ratios higher than 1∶10. The T_m_ at the limited molar ratio before condensation occurred was 91°C (Figure 4b). The Alba2 protein thermally stabilised AT-DNA (T°_m_, 62°C) by 7°C (T_m_, 69°C) at the molar ratio of 1∶2. The thermograms revealed two phase transitions at molar ratios above 1∶5 (Figure 5b). Similarly, as seen for AT-DNA, the thermal stabilisation of GC-DNA with Alba2 was greater compared to Alba1. At molar ratios higher than 1∶5, the T_m_ was shifted above 100°C. GC-DNA condensation was also seen at higher molar ratios (Figure 6b).

For the equimolar ratio of the Alba1/Alba2 proteins, the greatest effects on DNA thermal stability were with AT-DNA (Figure 5c). At the Alba1/Alba2:AT-DNA molar ratio of 1∶2, the shift in the T_m_ was 10°C. It is interesting to note that Alba1/Alba2 bind to AT-DNA at molar ratios of 1∶2, which is higher than that reported for genomic DNA.

### Analysis of UV Melting Curves

Our UV results clearly indicate that in the absence of the Alba proteins, the thermally induced helix-to-coil transitions of all of the DNAs studied occurred in a two-state manner. Assuming that the helix-to-coil transitions of Alba/DNA complexes at r = 0 and 0.2 can as a first approximation be considered as two-state transitions, except in the case of Alba2 binding to AT-DNA, the thermal stabilisation of the DNAs studied induced by the bound Alba1 and Alba2 can be, on the whole, discussed in terms of the formalism developed by [19]. The measured shifts in the melting temperature, T_m_, can be expressed as:
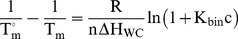
(2)where T^o^_m_ and T_m_ are the DNA melting temperatures in the absence and presence of the Alba proteins, respectively, ΔH_WC_ is the helix-to-coil transition enthalpy of the ligand-free duplex, which is 8.5 kcal/(mol bp) for calt thymus DNA, 7.6 kcal/(mol bp) for poly(dA-dT).poly(dA-dT), and 14.7 kcal/(mol bp) for poly(dG-dC).poly(dG-dC) [20]. Finally, n is the ratio of bound Alba proteins per DNA base pairs (0.2), and c is the equilibrium concentration of the Alba proteins, which can be estimated as one half of the total protein concentration. The calculated apparent binding constants, K_bin_, at T_m_ are listed in Table 1. The comparison of the K_bin_ values only separately for each DNA studied (in the same temperature range) can provide insight into the sequence specificity of the Alba proteins. There is no difference in the K_bin_ of Alba1 and Alba2 for CT-DNA, the K_bin_ of equimolar mixture of Alba1/Alba2 is in the same magnitude range. Alba2 has a higher K_bin_ for AT-DNA and GC-DNA than Alba1, but the K_bin_ is lower than for the equimolar mixture of Alba1/Alba2 at a molar ratio of 1∶5 (Table 1).

### Effects on the Secondary Structure of Alba1 and Alba2 Induced by Binding to DNA

**Figure 7:**
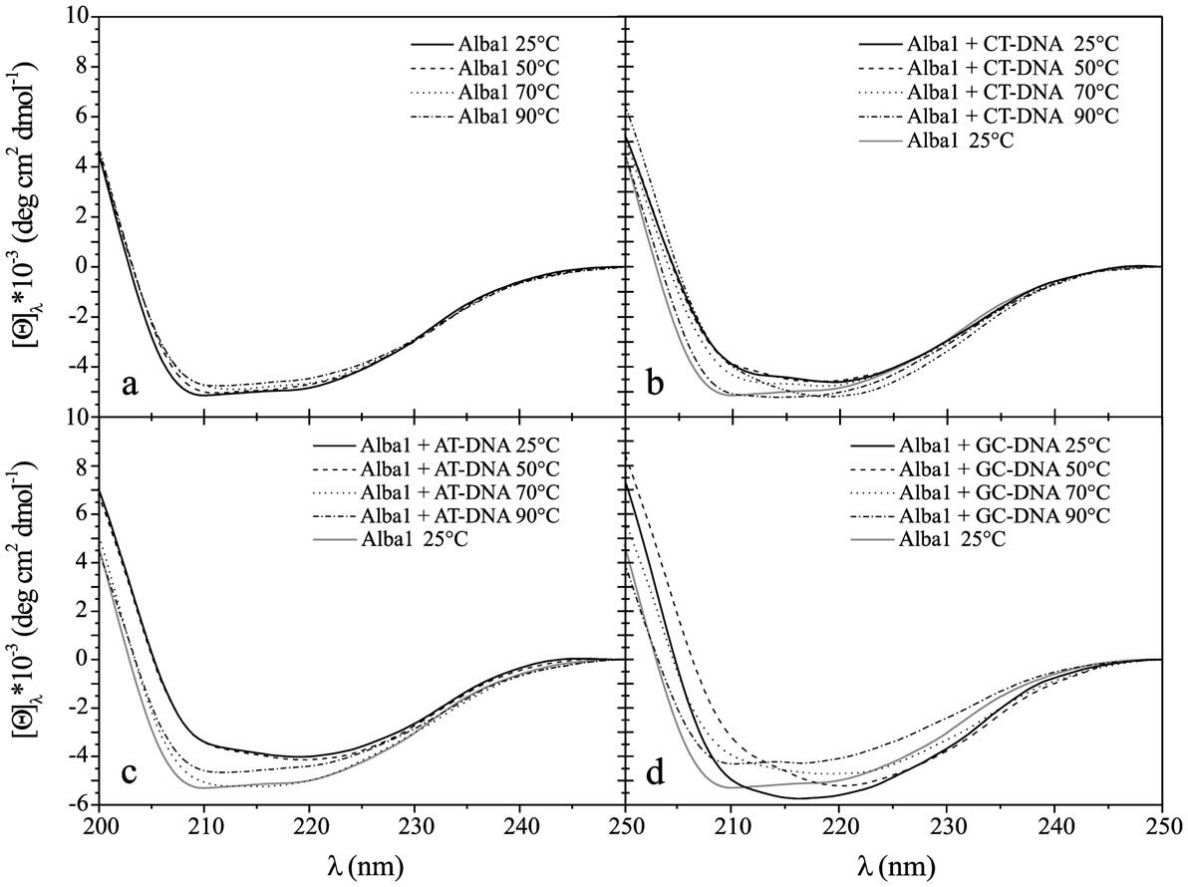
CD spectra of Alba1. Molar ellipticity, [Θ], in the far-UV range (200–250 nm) at different temperatures (as indicated), for Alba1 without bound DNA (a), with CT-DNA (b), with AT-DNA (c), and with GC-DNA (d). The molar ratio of Alba:DNA per base pair was 1∶5 at pH 7.0 (50 mM NaH_2_PO_4_).

Changes in the secondary structures of the Alba proteins that were induced by their binding to the various DNAs were followed by CD measurements in the far-UV CD range (200–250 nm). All of the spectra were measured at 25°C, 50°C, 70°C and 90°C. The secondary structures of the Alba proteins in *A. pernix* are similar to the Alba proteins in other hyperthermophiles, with alternating α, β structures (α1–β1–α2–β2–β3). The far-UV CD spectra of the Alba1 protein in the absence of the DNA oligonucleotides in the temperature range from 25°C to 90°C did not show any significant changes (Figure 7a). The estimated proportion of α-helices in Alba1 was *ca*. 22%, with β-sheets at 51% and β-turns at 27%. In the presence of CT-DNA, and at all temperatures (at a molar ratio of protein to DNA base pair of 1∶5), Alba1 adopted an apparent structure that had fewer α-helices and β-sheets, on account of increased amounts of non-periodic structure (21%). As the temperature was raised, the Alba1 protein again adopted a more ordered structure, with the most ordered structure reached at 90°C (Figure 7b). These changes in the structural organisation at higher temperatures might be the result of melting of the CT-DNA to single-stranded DNA, and thus dissociation of the Alba proteins from the dsDNA (Figure 4a). In the presence GC-DNA (at a molar ratio of 1∶5), the CD spectra of Alba1 differed from those in the presence of AT-DNA and CT-DNA (Figures 7b-d). The intensity of the CD spectra decreased (Figure 7d). The amount of β-structure increased at 50°C and 70°C. The GC-DNA (Figure 6a) remained in a double-stranded state, even at 90°C.

**Figure 8:**
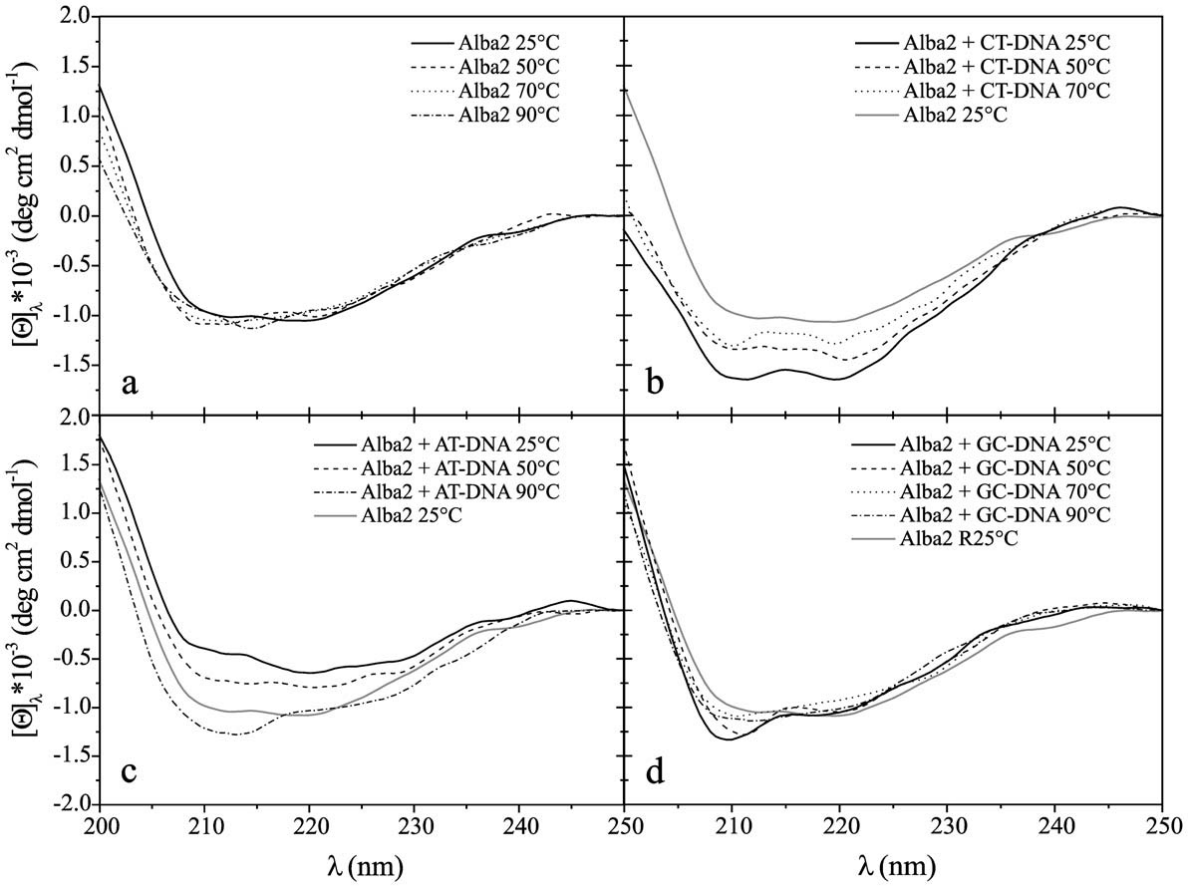
CD spectra of Alba2. Molar ellipticity, [Θ], in the far-UV range (200–250 nm) at different temperatures (as indicated), for Alba2 without bound DNA (a), with CT-DNA (b), with AT-DNA (c), and with GC-DNA (d). Molar ratio of Alba:DNA per base pair was 1∶5 at pH 7.0 (50 mM NaH_2_PO_4_).

The intensity of the far-UV CD spectra of Alba2 (Figure 8a) did not change significantly with temperature, as was observed for Alba1. The calculated amount of secondary structure elements for Alba2 from the CD spectra were 11% α-helices, 60% β-sheet, and 29% β-turns. These data would suggest that the Alba2 protein does not change in secondary structure according to temperature, in the temperature range studied. In the presence of CT-DNA (at a molar ratio of 1∶5) (Figure 8b), as with Alba1, Alba2 also adopted a more ordered structure as the temperature increased from 25°C to 90°C. In the presence of AT-DNA (at a molar ratio of 1∶5), the molar ellipticity of the far-UV CD spectra of Alba2 was lower, representing a less-ordered structure (Figure 8c), although the molar ellipticity increased with temperature. In the presence of GC-DNA (at the molar ratio of 1∶5), the far-UV CD spectra of Alba2 at 25°C showed spectral characteristics typical of a disordered structure. As the temperature increased, the structure became more ordered, with a higher content of β-strands. When the temperature approached 90°C, the structure became less ordered again, similar to that at 25°C (Figure 8d). Again, the explanation for these structural changes might be related to the equilibrium of dsDNA↔ 2× ssDNA (Table 1).

**Figure 9:**
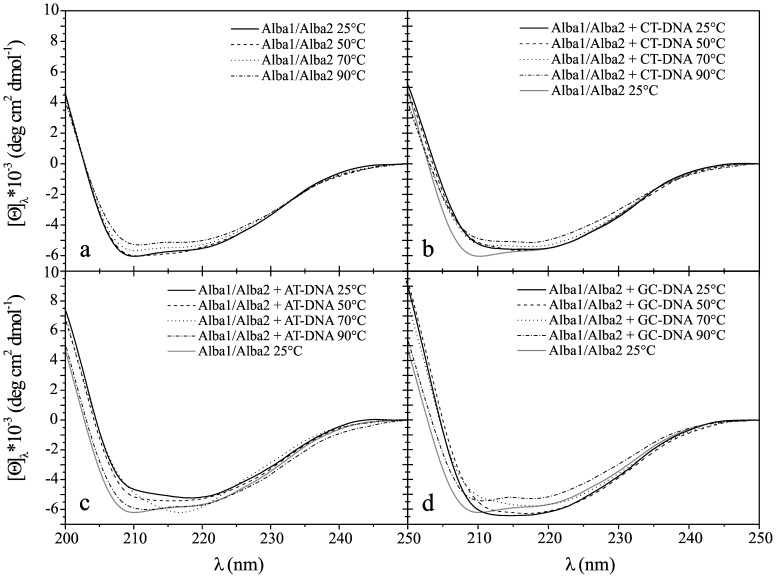
CD spectra of Alba1/Alba2 complex. Molar ellipticity, [Θ], in the far-UV range (200–250 nm) at different temperatures (as indicated), for the Alba1/Alba2 complex without bound DNA (a), with CT-DNA (b), with AT-DNA (c), and with GC-DNA (d). Molar ratio of Alba:DNA per base pair was 1∶5 at pH 7.0 (50 mM NaH_2_PO_4_).

The Alba1/Alba2 complex preserved its secondary structure content as the temperature was raised from 25°C to 70°C (25% α-helices, 48% β-sheet and 27% β-turns). As the temperature increased further, to 90°C, the amount of β-sheet increased to 61% at the expense of a decrease in the β-turn content, to 10% (Figure 9a). In the presence of CT-DNA, the equimolar mixture of Alba1/Alba2 underwent a slight change in secondary structure. As for each Alba protein measured separately, in the equimolar mixture Alba1/Alba2, a more ordered structure was gained at higher temperatures (with the most apparent increase seen in the β-sheet content). In the presence of AT-DNA and GC-DNA, Alba1/Alba2 did not trigger any significant changes in the conformation of the proteins (at 25°C), with the amount of β-sheet increasing with the increase in temperature (Figure 9 c, d).

This far-UV CD spectroscopy was used for the estimation of the secondary structure of the Alba proteins. As *A. pernix* is a hyperthermophile, a range of different temperatures was examined (25°C to 90°C). The estimated secondary structure of Alba1 (using the CONTIN software) in the absence of DNA did not change much with increasing temperature. In the presence of CT-DNA, the [Θ] and the shape of the spectra of Alba1 changed: the secondary structure of Alba1 became less organised at all of the temperatures, except at 90°C. The Alba1 protein had approximately the same level of structural organisation as at all of the temperatures in the absence of DNA. Similar results were obtained for Alba1 in the presence of AT-DNA. In the presence of GC-DNA, the [Θ] and the shape of the spectra of Alba1 was different. With increasing temperature, the [Θ] decreased. The β-loop levels, which are responsible for protein-DNA interactions, were 27% at 90°C in Alba1. Interestingly, only the addition of CT-DNA to Alba1 triggered a decrease in protein ordered structure (at 25°C to 70°C), on account of increases in other structures (15%).

### Isothermal titration Calorimetry

**Figure 10:**
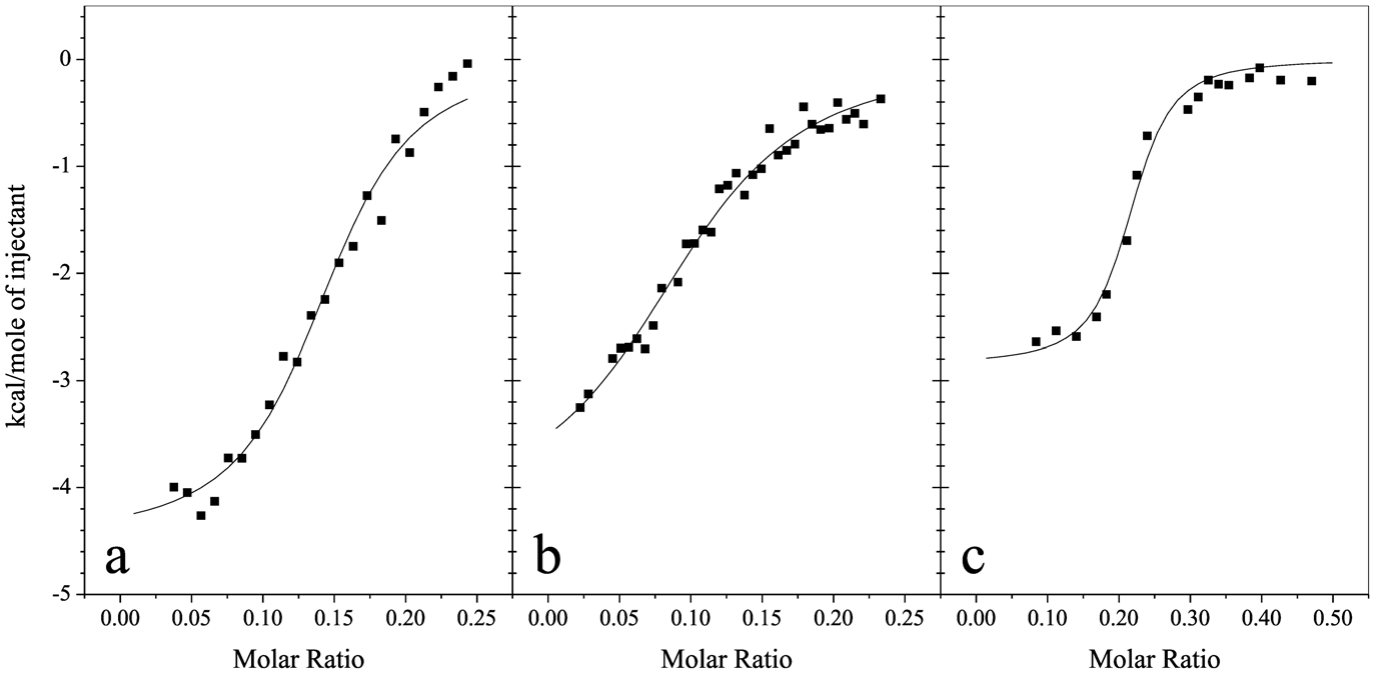
Isothermal titration calorimetry profiles of Alba1 binding to various DNA. A-DNA (a), and B-DNA (b) and CT-DNA (c), at 25°C in 50 mM NaH_2_PO_4_, pH 7.0.

The thermodynamic parameters of Alba1 binding to DNA were determined by ITC (Figure 10). The thermodynamic parameters obtained are given in Table 2, and include: binding stoichiometry (n); binding constant (K_bin_); ΔH_bin_; and TΔS_bin_. Detailed inspection of the thermodynamic data listed in Table 2 reveals that the binding constant, K_bin_, is the highest for CT-DNA, following by the A-DNA and the B-DNA. The binding of Alba1 to A-DNA has an enthalpically favourable ΔH_bin_, while entropically, the most favourable is the binding of Alba1 to CT-DNA. We could not measure the binding of Alba2 to these DNA oligonucleotides due to the condensation/precipitation, as the concentrations of the Alba proteins were higher for ITC than for the other methods studied here, due to the sensitivity limitations of the method.

**Table 2:** Thermodynamic profiles of Alba1 binding to the calf thymus DNA and DNA oligonucleotides at 25°C and pH 7.0.

DNA	n	K_bin_	ΔH_bin_	ΔS_bin_
		(10^6^ M^−1^)	(cal/mol)	(cal/mol K)
**CT-DNA**	0.21±0.01	7.8±2.2	−2849±112	22.0
**A-DNA**	0.15±0.01	2.4±0.5	−4477±167	14.2
**B-DNA**	0.11±0.01	0.8±0.1	−3112±492	17.9

n, binding site stoichiometry; K_bin_, binding constant; **Δ**H_bin_, enthalpy change of binding; ΔS_bin_, entropy change.

## Discussion

The majority of the sequenced archaeal genomes contain at least one gene encoding an Alba protein [21], making these probably the most conserved nucleic-acid-binding proteins in Archaea. It is believed that the Alba1 homodimer and Alba1/Alba2 heterodimer promote the organisation of chromatin at higher levels [4], [7], [8]; larger multimeric forms of the protein have not been reported [8], [22]. The ratio of the Alba1 and Alba2 proteins as potential homodimers and heterodimers in a cell at any specific moment is probably regulated by the physiological state of the cell, although Ssh10b in *Sulfolobus shibatae* is not affected by the cell cycle [23]. Native protein acrylamide electrophoresis reveals that the Alba1 protein from *Aeropyrum pernix* exists as a homodimer, in contrast to the Alba2 protein, which is in equilibrium between a monomeric and a dimeric state. Stronger Alba1 homodimerisation is probably the result of a disulphide bond between its Cys^47^ and the two neighbouring molecules; in contrast, an intramolecular disulphide bond between Cys^3^ and Cys^94^ of the Alba2 protein prevents formation of covalent dimers of two Alba2 proteins via this type of bond. The intramolecular disulphide bridge in Alba2 binds the β-loop L1 to the β3 sheet, which results in additional molecular stability [4]. Many studies, including our own, have clearly shown that Alba binds to double stranded DNA *in vitro*, although there has been some debate about its physiological role *in vivo* [24]. UV cross-linking data have suggested that an interaction with RNA might be more physiologically relevant. On the other hand, some studies have shown that Alba1 from *S. solfataricus* can stabilise dsDNA *in vivo*. It has also been shown that Alba1 binds dsDNA more tightly than either ssDNA or RNA *in vitro* [24], suggesting that it can differentiate between DNA and RNA.

Previously, it was reported that different Alba proteins bind with similar affinities to dsDNA fragments of different lengths [8]. Here, we applied the SPR technique to clarify whether there is a difference in the binding patterns for the Alba protein interactions with a dsDNA oligonucleotide: the SPRspecAP. The comparison of the SPR sensorgrams of the Alba1 and Alba2 proteins binding to SPRspecAP indicated that Alba1 is likely to bind with a higher binding rate, while more Alba2 can bind to the same DNA sequence. The SPR sensorgram of the Alba1/Alba2 complex showed a higher binding rate in the first part that resembles that of the Alba1 protein. Then the amount of complex stably bound to the SPRspecAP DNA sequence after the dissociation phase was comparable to that when only the Alba2 protein was bound to this DNA. The sensorgrams of the Alba1/Alba2 complex showed a more complex binding model. The SPR data analysis of Ssh10b indicated that the two forms of the Ssh10b dimer bind to the same DNA binding site, but have different conformational features that are responsible for the temperature-dependent nature of the Ssh10b–DNA interaction [25].

**Figure 11:**
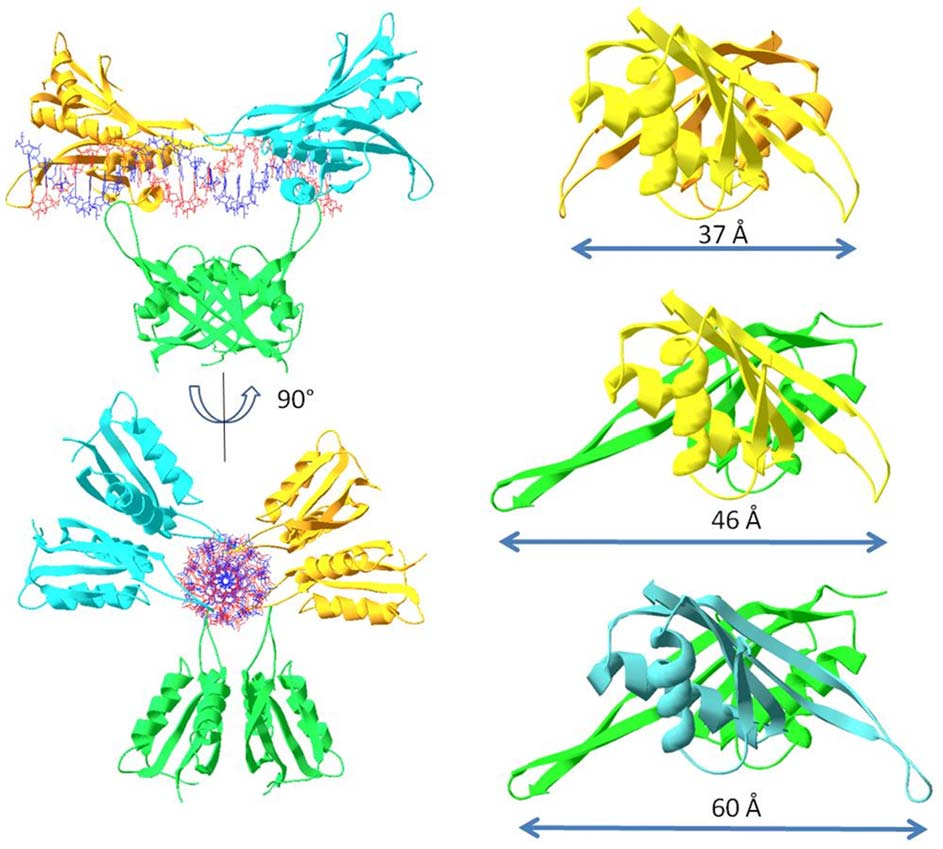
Molecular modelling of the Alba protein dimers. Left: Distances between the β3–β4 hairpins on the Alba1/Alba1 homodimer (top), the Alba1/Alba2 heterodimer (middle), and the Alba2/Alba2 homodimer (bottom). Right: Alba2 dimers covering the DNA duplex with maximal binding density of one dimer per six base pairs.

An interesting feature observed from the UV melting curves is that Alba2 thermally stabilises the AT-DNA, while the thermal stabilisation achieved by Alba1 was negligible. Similarly, the thermal stabilisation of the CT-DNA and GC-DNA was lower for Alba1 than for Alba2. On the other hand, the thermal stabilisation of all of these poly dsDNAs was greater for the equimolar mixture of both of these proteins (e.g., see Figures 4c, 5c, 6c). Furthermore, while Alba2 and the equimolar mixture of Alba1/Alba2 started to precipitate (condense) the GC-DNA oligonucleotide at a ratio of 1∶30, there was no such effect observed in the case of Alba1. This observation would suggest that Alba2 and the equimolar mixture of Alba1/Alba2 enhance the condensation/precipitation of CT-DNA and GC-DNA. Based on our data from the UV melting curves and calculated K_bin_ at melting temperature T_m_, we suggest that Alba2 and Alba1/Alba2 thermally stabilise AT-DNA at a greater extent than Alba1. As AT-rich regions are less stable than GC-rich regions in DNA, it appears physiologically sound to stabilise the regions that would otherwise melt at higher temperatures. The length of the β3–β4 hairpin is shorter for Alba1 than for Alba2. Therefore the span of DNA that can be reached by Alba1 is shorter than for Alba2, while the Alba1/Alba2 heterodimer lies between these two (Figure 11). This selection of different Alba hetero/homodimers provides the organism with a measure to accommodate different DNA duplexes that incorporate the features of A-DNAs or B-DNAs.

The changes in the secondary structure elements (determined from the far-UV CD spectra) indicate that the binding of Alba1 and Alba2 and the equimolar mixture of Alba1/Alba2 to the various DNAs (at the molar ratio of 1∶5 per base pair) is associated with noticeable changes in the secondary structure content of these proteins. Alba2 binds DNA with lower affinity than Alba1 [26]. Our ITC experiments indicate that Alba1 binds DNA, but we could not experimentally measure binding of Alba2 using this method. The gel shift [11] in addition to the ITC experiments, shows that Alba2 condenses DNA. Other than the UV melting curves that show some preferences of Alba2 for AT-DNA and GC-DNA, our data do not generally confirm that Alba2 is DNA-sequence specific. It is more likely that Alba2 and Alba1/Alba2 recognise specific conformational states of DNA.

It is known that Alba2 exists only as a heterodimer in the presence of Alba1, and that it affects DNA packaging [8]. An excellent study has just been published by Laurens and co-workers [27], where they show that Alba1 homodimers and Alba1/Alba2 heterodimers have distinct DNA-binding properties due to their difference dimer-dimer interactions. Alba1 binds cooperatively to single DNA molecules due to the F60 residue, which is not conserved in the Alba2 paralogue in *S. solfataricus*. The authors suggested that the dimer-dimer interface is responsible for two distinct structural effects on the DNA: bridging two DNA duplexes, and stiffening the DNA by cooperative side-by-side binding [24], [28]. The modes of bridging and binding stiffening depend on the protein:DNA stoichiometry. At low stoichiometry, Alba can bring two duplexes of DNA together; while under saturating conditions, Alba binds cooperatively along the DNA [24], [29]. In the case of the Alba1/Alba2 heterodimer, which lacks the conserved F60 residue at one side of the dimer, the dimer-dimer interactions along a single DNA duplex are limited to an interaction between two dimers [27]. The authors reported the condensation of DNA in the presence of the Alba1/Alba2 heterodimer without any stiffened configuration, as has been observed for the Alba1 homodimer [27].

The crystal structures of the Alba1 protein from several species have highlighted the conserved dimer–dimer interface [29]. In contrast, the *S. solfataricus* Alba2 protein is quite divergent in this region, as compared to Alba1, and this interface is not present in the crystal structure [24], [29]. The high resolution structure of the Alba2-dsDNA (16 bp) complex from *A. pernix* was recently reported [10]. The overall structure of the complex reveals a discrete mode of DNA binding, with the positively charged residues on the monomer-monomer interface of each dimer packing into the minor groove of the bound dsDNA, as was observed for Alba1 [29].

Phe60 is central to the dimer–dimer crystallographic interface of Alba1, where it forms a *π*–*π* stacking interaction with an adjacent dimer. As was shown by Jelinska and co-workers [24], the F60A mutantion of Alba1 decreases its binding affinity. This was consistent with the hypothesis that the dimer–dimer interface is involved in the assembly of Alba1–DNA nucleoprotein complexes, and suggests that additional protein–protein interactions formed upon binding of the wild-type protein to longer duplexes are not present in the F60A complexes. These data therefore support the assertion that a protein–protein interaction surface, similar to the crystallographic dimer–dimer interface, is involved in the assembly of Alba1 nucleoprotein filaments [24].

**Figure 12:**
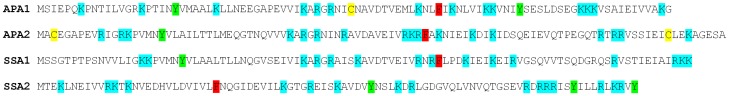
Sequence composition of the Alba proteins. The amino-acid sequences of Alba1 (APE1832.1–APA1) and Alba2 (APE1823–APA2) from *A. pernix* was aligned with those of the homologous proteins from *Sulfolobus shibitae* Alba1 (P60849– SSA1) and *S. solfataricus* Alba2 (Q97ZF4–SSA2). The positively charged amino acids (R and K), phenylalanine (F), tyrosine (Y) and cysteine (C) are marked.

Alba2 is expressed at 5% to 10% of the Alba 1 protein levels [8]. The fact that Alba2 forms heterodimers with Alba1, which behave similarly to Alba2 based on our SPR and UV melting curves (thermal stabilisation of AT sequences, precipitation of GC sequences), and differently than Alba1 might provide a mechanism for the control of chromatin packaging in this organism, as was suggested for the Alba1/Alba2 heterodimer from *S. solfataricus* [8], [24]. However, a functionally crucial difference between the *S. solfataricus* Alba1 and Alba2 proteins is the presence of an F60 residue in Alba1, which is not conserved in Alba2, while in Alba2 from *A. pernix*, a phenylalanine is at position 58 (Figure 12). It appears that the *Aeropyrum* homologues both contain the equivalent of this residue, and are thus both Alba1-type proteins. This might mean that potential heterodimer formation does not have such distinct effects on DNA binding as seen with the *Sulfolobus* proteins [24], [27].

The high resolution structure of the Alba2–dsDNA complex from *A. pernix* revealed the binding of only a small segment of dsDNA close to the Alba2 dimer interface [10]. It is possible that the short segment of DNA used for the crystallisation prevented the binding of several Alba2 dimers to the DNA, and its crystallisation. Our docking of the Alba dimer to the B-DNA (ACGTACGTACGTACGTACGTACGTACGTACGTACGTACGTACGTACGT) provided a solution similar to that reported previously by Wardleworth and co-workers [29]. Based on this type of docking conformation, the plausible solution to achieve a high density of Alba dimer binding (as one dimer per six base pairs) is that the next consecutive Alba dimer binds to the DNA rotated by approximately one third of a full turn from the previously bound dimer, therefore avoiding the steric clash proposed previously by Wardleworth et al. [29]. The separation between the consecutive dimers can be smaller for the Alba1 and Alba1/Alba2 dimers, which span shorter distances and can bind to a DNA conformation that has a smaller periodicity, e.g. the A-DNA (CCCGGGCCCGGGCCCGGGCCCGGGCCCGGGCCCGGGCCCGGGCCCGGG).

The ability of Alba proteins to bridge two DNA duplexes suggests an important role in shaping archaeal cromatin structure. Architectural proteins that bridge DNA allow the formation of loops, which can functionally organise the genome [30]. As Alba2 is expressed only at a few percent of Alba1, the majority of Alba *in vivo* will be in the form of Alba1 homodimers. Previous studies have shown that small positively charged chromosomal proteins, such Sul7 and Cren7, can bind non-specifically to the DNA minor groove and sharply kink duplex DNA via intercalation [31]. In *A. pernix*, a similar small basic protein, CC1 (Ape1322b), has been found [32], which provides significant thermally stabilisation of DNA [33] in comparison to the Alba proteins, as we have seen here. It is likely that these small basic proteins in Crenarchaea are involved in the bending/kinking of the DNA to pack the chromosomal DNA more tightly in the cell, as it has recently been shown for the Cren7 and Sul7 proteins [34].
